# Study on the physiological and metabolic mechanisms of exogenous quercetin in cadmium hyperaccumulator *Amaranthus hypochondriacus* L

**DOI:** 10.3389/fpls.2026.1788620

**Published:** 2026-04-14

**Authors:** Yang Zhou, Yingying Gui, Yuling Liu, Qiang Zhou, Cheng Qiu, Dagang Song

**Affiliations:** 1 State Key Laboratory of Water Engineering Ecology and Environment in Arid Area, Xi’an University of Technology, Xi’an, China; 2 School of Architecture and Civil Engineering, Chengdu University, Chengdu, Sichuan, China; 3 School of Computer, Chengdu University, Chengdu, Sichuan, China; 4 Department of Material and Environmental Engineering, Chengdu Technological University, Chengdu, Sichuan, China; 5 Biogas Institute of Ministry of Agriculture and Rural Affairs, Chengdu, China

**Keywords:** *Amaranthus hypochondriacus* L., antioxidant, cadmium stress, flavonoid metabolism, quercetin, stress resistance

## Abstract

**Introduction:**

Improving cadmium (Cd) tolerance and phytoremediation efficiency in hyperaccumulator plants is a critical scientific issue in environmental remediation. Remediation performance is often constrained by physiological bottlenecks, including insufficient tolerance to high Cd stress and low heavy metal accumulation capacity. Against this background, exploring effective strategies to enhance the phytoremediation efficiency of hyperaccumulators has important theoretical and practical value.

**Methods:**

Using *Amaranthus hypochondriacus* L. (amaranth) cultivar R104, quercetin (0, 5, 10, 20 mg·L^-1^; denoted as CK, Q1, Q2, and Q3, respectively) was applied under varying Cd levels (0, 4, 20 mg·kg⁻¹; denoted as Cd0, Cd4, and Cd20, respectively), and physiological traits, antioxidant responses, and key metabolites were comprehensively assessed.

**Results:**

The results demonstrate that exogenous quercetin markedly alleviated Cd induced toxicity, with the Cd20Q2 treatment showing the most pronounced mitigation effect. Compared the Cd stressed control without quercetin, electrolyte leakage was reduced by 49.5%, chlorophyll content increased by 17.7%, and the plant’s Cd enrichment capacity was significantly enhanced, with the aboveground enrichment factor reaching 7.02. It revealed that quercetin activated the phenylpropanoid flavonoid pathway, promoting the synthesis of endogenous flavonoids (notably quercetin and kaempferol) and increasing glutathione (GSH) levels and overall antioxidant capacity. This created a synergistic mechanism of “endogenous flavonoid enhancement coupled with exogenous quercetin supplementation“. Concurrently, the enhanced accumulation of GSH and related metabolites facilitated Cd chelation and detoxification, thereby reducing oxidative injury at the cellular level.

**Discussion:**

In summary, exogenous quercetin improves Cd tolerance and remediation efficiency in amaranth by regulating flavonoid metabolism and strengthening GSH mediated detoxification. These findings provide theoretical and practical support in heavy metal remediation strategies.

## 1 Introduction

With the rapid development of modern industry and agriculture, heavy metal cadmium (Cd) pollution has become a global environmental issue (Li et al., 2017; Mohamed et al., 2025; Sidhu et al., 2020). Its high mobility and persistence characteristics not only lead to soil fertility decline, but also cause microbial community imbalance and reduced crop yields. Furthermore, through bioaccumulation, it causes irreversible damage to the liver, kidneys, bones, and cardiovascular systems of animals, plants, and humans, severely threatening ecological safety, agricultural product quality, and public health (Hu et al., 2025). Among various Cd contamination remediation techniques, phytoremediation stands out. It has become the mainstream *in situ* remediation method due to its core advantages, including low cost, environmental friendliness, and no secondary pollution. Heavy metal stress often triggers a rapid accumulation of reactive oxygen species (ROS) in plants, leading to membrane lipid peroxidation, impaired photosynthetic systems, and consequently reduced biomass and remediation efficiency. To cope with Cd toxicity, plants adopt multiple defense strategies, including cell wall immobilization, vacuolar sequestration, ROS scavenging by flavonoids, and heavy metal chelation via phytochelatins (PCs) (Srivastava et al., 2014). Across these processes, thiol containing compounds play a central role in Cd binding and detoxification. Cd stress disrupts cellular metabolism, induces excessive ROS generation, and triggers lipid peroxidation and oxidative damage, ultimately compromising membrane integrity and increasing electrolyte leakage (Lotfipour Nasudivar et al., 2024). In response, amaranth enhances both enzymatic and non-enzymatic antioxidant systems to scavenge ROS and alleviate oxidative injury. Amaranth activates detoxification pathways that sequester absorbed Cd into vacuoles, thereby reducing its direct cytoplasmic toxicity and reinforcing metal fixation and compartmentalization mechanisms. Among non-enzymatic antioxidants, small molecules such as GSH, ascorbic acid, and flavonoids are particularly critical (Bellini et al., 2023; Li et al., 2021). GSH, containing a thiol group, directly scavenges free radicals, participates in the glutathione-ascorbate (GSH-AsA) cycle to maintain redox homeostasis, and plays an essential role in Cd chelation and detoxification (Luo et al., 2024). The phenylpropanoid flavonoid pathway, responsible for flavonoid biosynthesis, is also activated under Cd stress. Its downstream metabolites enhance ROS scavenging and help maintain cellular structural stability, thereby enabling greater GSH availability for Cd chelation (Kumari et al., 2025; Sreelatha et al., 2025).

Amaranth, a highly adaptable species with high biomass and rich nutritional value, has been identified as a Cd hyperaccumulator and has shown considerable potential for the remediation of heavy metal contaminated soils in recent years (Ghuge et al., 2023; Han et al., 2023; Xie et al., 2023). Previous studies have shown that both the seeds and leaves of amaranth exhibit notable antioxidant activity, effectively inhibiting 2,2-diphenyl-1-picrylhydrazyl (DPPH) radical formation, with leaf tissues displaying stronger antioxidant capacity than seeds (López-Mejía et al., 2014). Some researchers have found that amaranth exhibits good Cd accumulation in various soils, but the accumulation efficiency is significantly influenced by the cation exchange capacity (CEC) of the soil (Cui et al., 2021). Although amaranth exhibits a strong response to Cd, single plant remediation technologies still face challenges of low remediation efficiency. Enhanced phytoremediation was actively investigated. In recent years, numerous scholars have conducted extensive studies on enhancing phytoremediation, with abundant literature demonstrating the optimization effects achieved through microorganisms, biochar, genetic engineering, exogenous additives, etc (Bhat et al., 2022; Wang et al., 2020). Research on exogenous additives has become a major focus in enhancing phytoremediation efficiency. Plant growth regulators (PGRs) have been widely applied in the remediation of Cd contaminated soils using amaranth. Foliar application of indole-3-butyric acid (IBA), diethyl aminoethyl hexanoate (DA-6), 24-epibrassinolide, and 2,4-dichlorophenoxyacetic acid (2,4-D) has been reported to effectively promote Cd accumulation in the aboveground tissues of amaranth (Faseela et al., 2025; Sehrish et al., 2024; Xie et al., 2020; Yang et al., 2024). Other commonly used exogenous additives include ethylenediaminetetraacetic acid (EDTA), organic acids, and gibberellins. Wang Kai further investigated multiple chelating agents and their combined applications, found that they significantly enhanced the Cd remediation efficiency of amaranth (Wang et al., 2019). However, many of these additives pose potential risks of secondary environmental pollution (Le et al., 2020).

Flavonoids have attracted increasing attention as environmentally friendly additives because of their strong ability to enhance plant stress resistance (Shen et al., 2022). Exogenous flavonoids (e.g., quercetin and rutin) possess multiple bioactivities, including strong antioxidant effects, heavy metal chelation, and the regulation of plant stress resistance metabolism (Shomali et al., 2022). Studies have shown that rutin application under heavy metal stress can alleviate Cd toxicity, significantly increase plant biomass and antioxidant capacity, and simultaneously elevate the levels of flavonoid secondary metabolites (Gorni et al., 2022; Kang et al., 2022). Quercetin, one of the widely distributed natural flavonoids in nature, has gained significant attention in recent years for its role in alleviating plant heavy metal stress (Carrillo-Martinez et al., 2024; Batiha et al., 2020; Singh et al., 2021). Studies have confirmed that exogenous quercetin can mitigate the toxic effects of heavy metals in plants by activating the antioxidant enzyme system and promoting the synthesis of metal chelating peptides (Jańczak-Pieniążek et al., 2022a; Migut et al., 2022; Zhang et al., 2024). However, the specific physiological response patterns and metabolic regulatory pathways of exogenous quercetin in amaranth have not been systematically explored. How exogenous quercetin targets and regulates the Cd tolerance, accumulation, and translocation efficiency in amaranth, and whether it enhances remediation potential by restructuring key metabolic pathways such as flavonoid synthesis and Cd chelation, remain unexplored. The lack of research on these scientific questions limits the understanding of the interaction between exogenous bioactive substances, hyperaccumulating plants and heavy metals at both physiological and metabolic levels, thereby hindering the development of efficient plant-assisted remediation technologies.

Therefore, this study is to elucidate the metabolic mechanisms underlying quercetin-enhanced Cd remediation, focusing on key metabolites in the phenylpropanoid flavonoid and glutathione-phytochelatins (GSH-PCs) pathways, and to assess the impact of quercetin on Cd tolerance and accumulation efficiency. This work is expected to deepen the understanding of the interaction between exogenous bioactive compounds and hyperaccumulator plants under heavy metal stress, and to provide a theoretical foundation for improving the efficiency of phytoremediation of Cd contaminated soils.

## 2 Materials and methods

### 2.1 Experimental materials

Amaranth cultivar R104 seeds were provided by Hunan Agricultural University. Amaranth R104 is a proven Cd hyperaccumulator genotype with stable Cd accumulation capacity and strong adaptability, which has been widely used in related studies on Cd phytoremediation (Xie et al., 2020). Selecting this genotype has ensured the reliability and repeatability of the experimental results. The experiment was conducted in a greenhouse located in Shuangliu District, Chengdu City, Sichuan Province, China. Surface soil sample were collected from a local agricultural field. The soil possessed uniform texture and stable physicochemical characteristics, providing a stable and homogeneous growth substrate for Amaranth. After collection, visible plant residues and roots were removed. The soil was then dried in air at room temperature for one month, ground using a mortar, and passed through a sieve before further analysis. The physicochemical properties of the soil were determined and are presented in **Table 1** (Orzoł et al., 2022; Pierzynski and Logan, 1993; Kempers and Zweers, 1986; Meersmans et al., 2009; Rab et al., 2011; Kalra, 1995).

**Table 1:** Soil basic properties.

Soil parameter	Value
Cd	0.306 mg·kg^-1^
Available phosphorus	1.46 mg·kg^-1^
Ammonia nitrogen	51.83 mg·kg^-1^
Organic matter	1.34%
Field capacity	629 g·kg^-1^
pH	6.1

### 2.2 Experimental design

Cd4and Cd20 soils were prepared by uniformly spraying Cd chloride solution onto the soil surface according to calculated dosages, followed by thorough mixing. After homogenization, urea, potassium dihydrogen phosphate, and potassium nitrate were added as basal fertilizers. The amended soils were allowed to equilibrate for one month. Amaranth seeds were germinated in seedling trays under greenhouse conditions (average temperature ~20 °C) for 14 days. When seedlings reached approximately 5 cm, healthy and uniformly sized individuals were transplanted into plastic pots (50 cm × 20 cm × 18 cm), each containing 10 kg of soil. The experiment was arranged in a completely randomized design with a factorial combination of soil Cd levels (0, 4, 20 mg·kg^-1^; denoted as Cd0, Cd4, and Cd20, respectively) and quercetin concentrations (0, 5, 10, 20 mg·L^-1^; denoted as CK, Q1, Q2, and Q3, respectively), resulting in 12 treatments. Each treatment included three independent pots (n = 3), and each pot (10 kg soil) was considered the experimental unit, as shown in **Table 2**. After transplanting, five uniformly sized seedlings were maintained per pot. Pots were randomly assigned to greenhouse bench positions using random numbers, and their positions were rotated weekly to minimize potential positional effects. Plants were grown in a greenhouse under natural light; temperature was maintained at approximately averaged 21 °C. All pots were irrigated with ultrapure water to maintain soil moisture at approximately 60% field capacity, and identical water and nutrient management was applied across treatments. Quercetin stock solution (1 mg·mL^-1^) was prepared in absolute ethanol and sonicated in an ice water bath for 30 min until completely dissolved. The stock was then serially diluted with ultrapure water to obtain working solutions. The ethanol concentration applied is consistent across all groups. All solutions were freshly prepared and protected from light prior to use. Forty-five days after transplanting, quercetin solutions were sprayed onto both sides of the leaves until runoff; control groups received the same volume of water. A total of 6 applications were made: twice daily spray applications of 500 mL each on days 45, 49, and 53 post transplantation. Harvest was conducted 60 days after transplanting. For non-destructive traits, three plants per pot were measured and averaged, whereas for destructive and biochemical analyses, one plant per pot was sampled at harvest.

**Table 2:** Experimental treatment design.

C_Exogenous quercetin_	C_Exogenous Cd_		
	Cd (0 mg·kg^-1^)	Cd (4 mg·kg^-1^)	Cd (20 mg·kg^-1^)
CK (0 mg·L^-1^)	Cd0CK	Cd4CK	Cd20CK
Q1 (5 mg·L^-1^)	Cd0Q1	Cd4Q1	Cd20Q1
Q2 (10 mg·L^-1^)	Cd0Q2	Cd4Q2	Cd20Q2
Q3 (20 mg·L^-1^)	Cd0Q3	Cd4Q3	Cd20Q3

### 2.3 Plant height and biomass measurement

Randomly collected 3 plants of amaranth per group. Measure plant height with a tape measure. Rinse roots with tap water, then wash entire plant with ultrapure water and pat dry. Weigh and record as fresh weight. Dry these plants in a 60 °C oven until constant weight is reached, indicating complete moisture evaporation. The final stable weight is recorded as dry weight.

### 2.4 Electrolyte leakage

Fresh leaves (1 g) were washed and cut into 1 cm segments. Samples were incubated in 10 mL ultrapure water at 35°C for 1 h, and initial conductivity (EC1) was measured. Tubes were then autoclaved at 121°C for 20 min and cooled to room temperature for final conductivity (EC2) (Dionisio-Sese and Tobita, 1998). EL was calculated as:


$$EL\left(\%\right)=\frac{EC1}{EC2}\times 100$$

### 2.5 Chlorophyll content

A handheld chlorophyll meter (Zhongkewei Science & Technology Co, China) was used to measure relative chlorophyll content, expressed as Soil Plant Analysis Development (SPAD) value based on transmitted light intensity. The SPAD value was calculated using (Zhang et al., 2022):


$$SPAD=A\times lg\left(\frac{I_{R}}{T_{R}}\right)-B\times lg\left(\frac{I_{NIR}}{T_{NIR}}\right)$$

Where, A and B are calibration coefficients.

I_R_: The intensity of incident red light.
I_NIR_: The intensity of incident near-infrared light.
T_R_: Red light intensity after passing through the leaves.
T_NIR_: Near-infrared light intensity after passing through the leaves.

### 2.6 Total antioxidant capacity and glutathione content

Tissue samples (g) were homogenized in an ice bath using extraction solution at a 1:10 volume ratio (approximately 0.1 g tissue weighed and mixed with 1 mL pre-chilled extraction solution) (Alpert and Gilbert, 1985; Pellegrini et al., 2006). The mixture was then centrifuged at 10,000 rpm for 10 minutes at 4 °C and stored frozen for analysis. The supernatant from the homogenate was tested using the kit with a UV spectrophotometer.

### 2.7 Cd content determination

Plant samples were dried to constant weight after collection by organ, pulverized using a grinder, sieved through a 100 mesh screen, and then digested using the hot plate method before measurement by inductively coupled plasma mass spectrometry (ICP-MS) (Orzoł et al., 2022). Reagent blanks and the standard reference material (GBW10015a, spinach) were digested and analyzed using the same procedures for quality control and assurance, with Cd recoveries in plant tissues ranging from 85% to 105%.

### 2.8 Flavonoid and phenolic acid content

Cinnamic acid and p-coumaric acid content in samples were determined by high performance liquid chromatography (HPLC) following the method of Nour et al. (2013) with results expressed as μmol·g^−1^ fresh weight (FW), while kaempferol and quercetin content were measured using the method of Olszewska (2008), with results expressed as μg·g^−1^ FW. Total flavonoid content was determined using the sodium nitrite-aluminum nitrate colorimetric method and measured by UV spectrophotometry, with results expressed as μg·g^−1^ FW (Chang et al., 2002). **Table 3** presents the calibration data and analytical parameters for kaempferol, quercetin, and total flavonoids, including the standard curve equations, R^1^ values, recovery rates, and limits of detection (LOD) and quantification (LOQ). These results demonstrate the accuracy and reliability of the analytical methods used for flavonoid quantification.

**Table 3:** Calibration data and analytical parameters for flavonoids.

Determined indicators	Standard curve equation	R^2^	Recovery rate	LOD	LOQ
kaempferol	y = 189.43x+10.804	0.9902	96.27%-102.15%	0.38	1.17
Quercetin	y =103.68x+18.403	0.9913	91.33%-101.08%	0.46	1.66
Total flavonoids	y = 0.7848x+0.0394	0.9994			

### 2.9 Phenylalanine content

Approximately 0.1 g of tissue sample was weighed, dissolved in 5 mL of 80% methanol (v/v), and subjected to ultrasonic extraction for 30 min. The mixture was then centrifuged at 4,000×g for 10 min. The supernatant was evaporated to dryness, redissolved in 1 mL of methanol, and filtered through a 0.22 μm microporous membrane. An appropriate amount of the filtrate was subjected to o-phthalaldehyde (OPA) derivatization. After reacting at room temperature for 2 min, inject immediately for analysis. High performance liquid chromatography-diode array detection (HPLC-DAD) system was employed with a C18 column (250 mm × 4.6 mm, 5 μm). The mobile phase consisted of A (0.1% formic acid aqueous solution) and B (methanol) at a flow rate of 1.0 mL·min^−1^. Column temperature was maintained at 30 °C with an injection volume of 10 μL. The detection wavelength was set at 338 nm, corresponding to the maximum absorption peak of the OPA-phenylalanine derivative. A series of standard solutions with varying concentrations was prepared using phenylalanine standard samples derivatized under identical conditions. A standard curve was established to calculate the phenylalanine content in the samples (Fontes et al., 2024), with results expressed as μmol·g^−1^ dry weight (DW).

### 2.10 Data analysis

All data in this paper were collected and organized using Excel, plotted using Origin and Chiplot, and analyzed using IBM SPSS Statistics 27 for univariate analysis, multiple comparisons, and correlation analysis.

Formula for calculating the aboveground enrichment coefficient (Ghosh and Singh, 2005):


$$EF_{shoot}=\frac{C_{shoot}}{C_{soil}}$$

## 3 Results

### 3.1 Effects of exogenous quercetin on the growth of *Amaranthus hypochondriacus* L. under cadmium stress

#### 3.1.1 Effects on plant biomass

**Figure 1:**
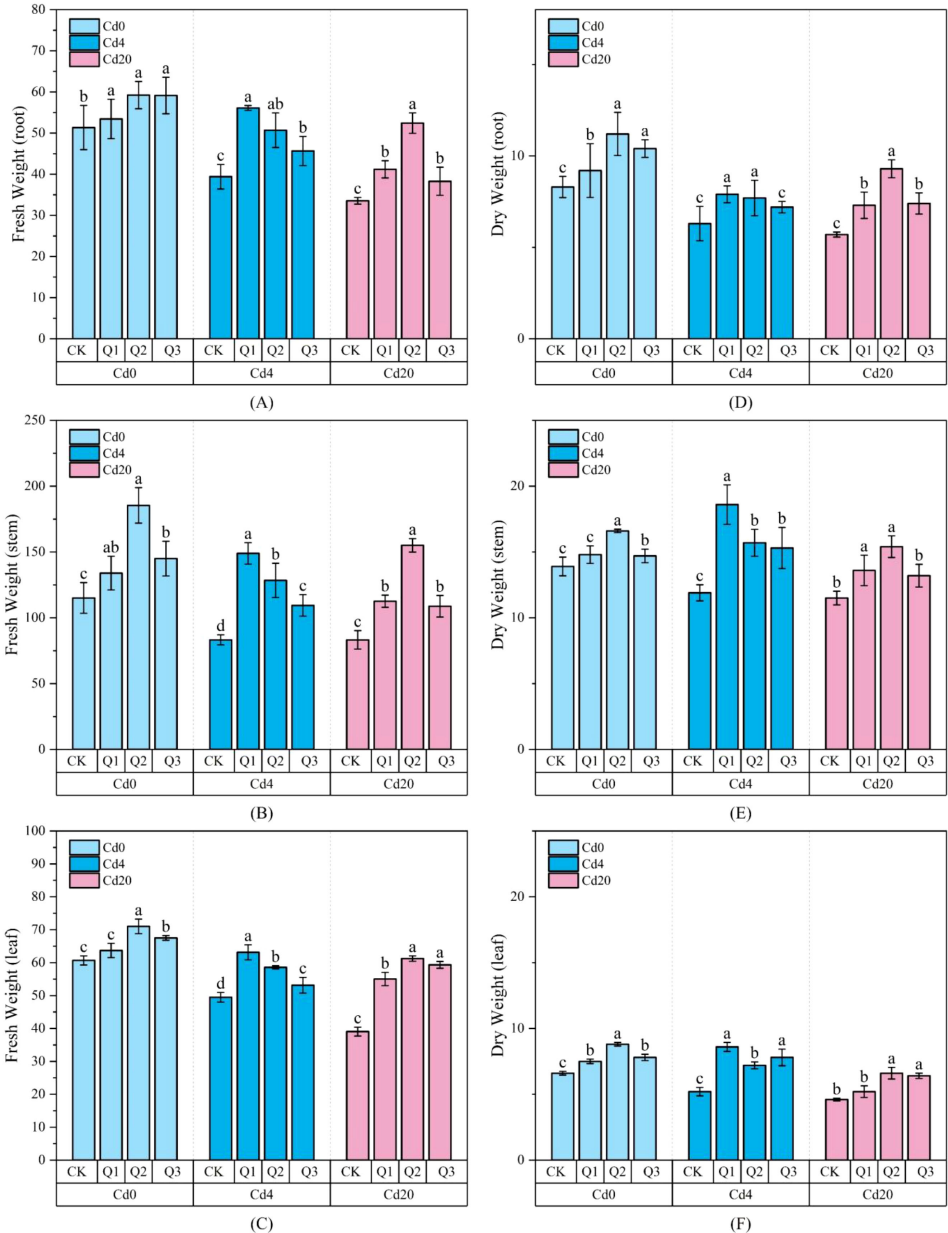
Fresh and dry weights (g) of amaranth under different treatment conditions: **(A)** root fresh weight; **(B)** stem fresh weight; **(C)** leaf fresh weight; **(D)** root dry weight; **(E)** stem dry weight; **(F)** leaf dry weight. Values are expressed as mean ± SD (standard deviation) (n=3). Bars with different letters differed significantly (*p* < 0.05) among the experimental groups. The experiment included three Cd treatment levels (Cd0 = 0 mg·kg^-1^, Cd4 = 4 mg·kg^-1^, Cd20 = 20 mg·kg^-1^) and four quercetin application concentrations (CK = 0 mg·L^-1^, Q1 = 5 mg·L^-1^, Q2 = 10 mg·L^-1^, Q3 = 20 mg·L^-1^).

In **Figure 1**, Cd stress significantly inhibited fresh weight accumulation in amaranth. Both fresh and dry weights of roots, stems, and leaves decrease exhibited a decreasing trend with increasing Cd concentration (Cd0 > Cd4 > Cd20). This indicates that Cd stress negatively impacts plant growth across all organs, with the roots being the first to be affected due to direct interaction with Cd in the soil. As Cd moves upward through the plant, it inhibits stem and leaf growth, likely due to oxidative stress and disruptions in essential metabolic processes. Overall, this suggests that Cd adversely affects plant growth in a systemic manner, impeding biomass accumulation and Cd translocation, which could limit the plant’s potential for phytoremediation. In the Cd0 group, overall growth status was good. Under Cd4 and Cd20 stress, total fresh weight decreased by approximately 24.21% and 31.37%, respectively, while dry weight decreased by 12.59% and 24.49%. This indicates that Cd toxicity significantly inhibited the growth of amaranth. Exogenous quercetin application markedly alleviated the decline in fresh weight under Cd stress (*p* < 0.05), with the most pronounced effect observed in the Cd20Q2 treatment (10 mg·L^-1^). In the Cd4 group, fresh weight increased to 268.12 g and dry weight to 36.35 g under Q1 treatment, representing approximately 29.61% and 50.01% increases compared to the untreated CK group. Similarly, the Cd20 group achieved maximum fresh weight (268.72 g) and dry weight (31.16 g) under Q2 treatment, representing increases of 72.43% and 47.96% over the control. However, the Q3 treatment (excessively high concentration) showed a slight decrease, indicating that quercetin’s promotion of fresh weight in amaranth is concentration dependent. Moderate concentrations can most effectively alleviate growth inhibition caused by Cd stress.

#### 3.1.2 Effects on chlorophyll content

**Figure 2:**
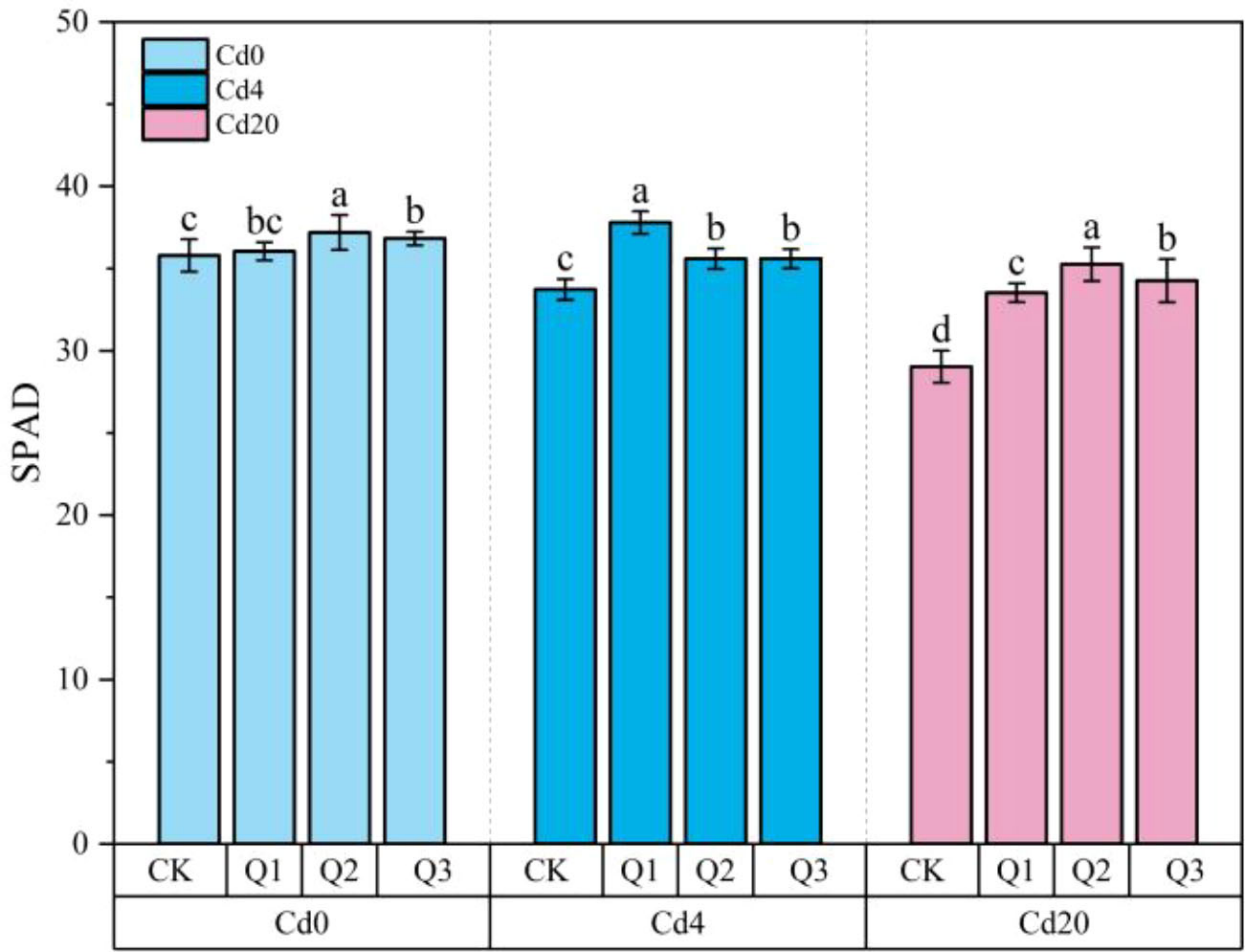
Changes in SPAD values under different treatment conditions. Values are expressed as mean ± SD (n=3). Bars with different letters differed significantly (*p* < 0.05) among the experimental groups. The experiment included three Cd treatment levels (Cd0 = 0 mg·kg^-1^, Cd4 = 4 mg·kg^-1^, Cd20 = 20 mg·kg^-1^) and four quercetin application concentrations (CK = 0 mg·L^-1^, Q1 = 5 mg·L^-1^, Q2 = 10 mg·L^-1^, Q3 = 20 mg·L^-1^).

Chlorophyll is a key indicator of plant photosynthetic performance, and variations in SPAD values can reflect the extent of photosynthetic limitation and stress induced damage. As shown in **Figure 2**, SPAD values declined progressively with increasing Cd concentration (Cd0 > Cd4 > Cd20), indicating that Cd stress impaired chlorophyll synthesis and stability in amaranth. Exogenous quercetin application (Q1, Q2, Q3) significantly enhanced leaf chlorophyll content across all Cd levels (*p* < 0.05). In the Cd20 group, SPAD values increased by 15.50%, 22.85%, and 18.03% under Q1, Q2, and Q3, respectively, markedly surpassing the control (Cd20CK). These results suggest that quercetin effectively mitigates Cd induced oxidative damage thereby improving chlorophyll retention and photosynthetic performance in amaranth under Cd stress.

### 3.2 Effects of exogenous quercetin on cadmium accumulation capacity in different organs of *Amaranthus hypochondriacus* L. under cadmium stress

#### 3.2.1 Comparison of cadmium accumulation capacity in different organs

**Figure 3:**
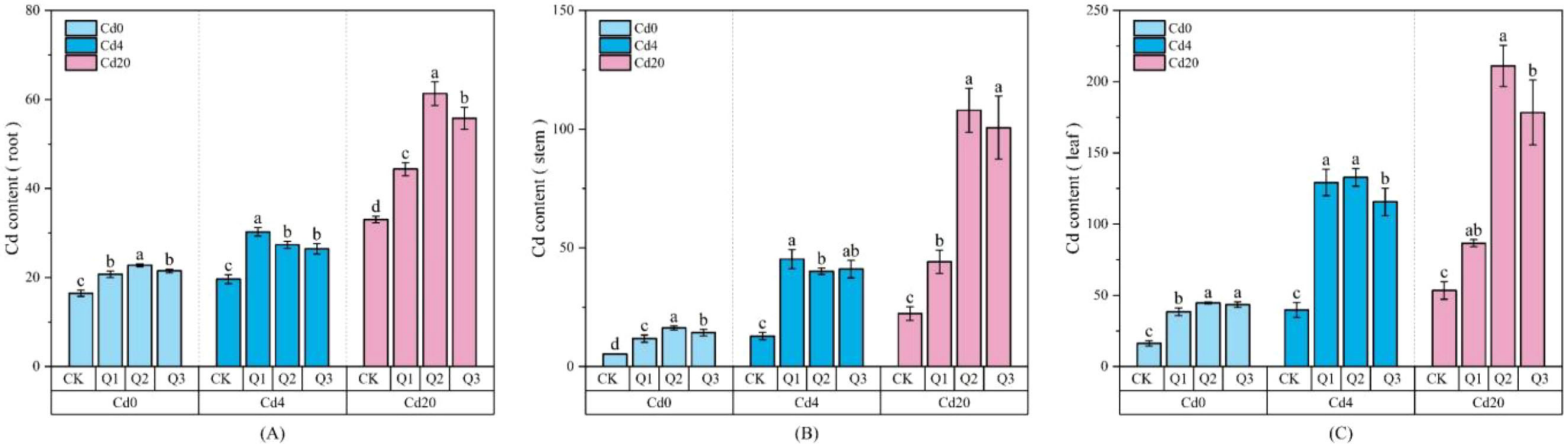
Cd accumulation in roots, stems, and leaves of amaranth under different treatment conditions: **(A)** Cd accumulation in root; **(B)** Cd accumulation in stem; **(C)** Cd accumulation in leave. Values are expressed as mean ± SD (n=3). Bars with different letters differed significantly (*p* < 0.05) among the experimental groups. The experiment included three Cd treatment levels (Cd0 = 0 mg·kg^-1^, Cd4 = 4 mg·kg^-1^, Cd20 = 20 mg·kg^-1^) and four quercetin application concentrations (CK = 0 mg·L^-1^, Q1 = 5 mg·L^-1^, Q2 = 10 mg·L^-1^, Q3 = 20 mg·L^-1^).

**Figure 4:**
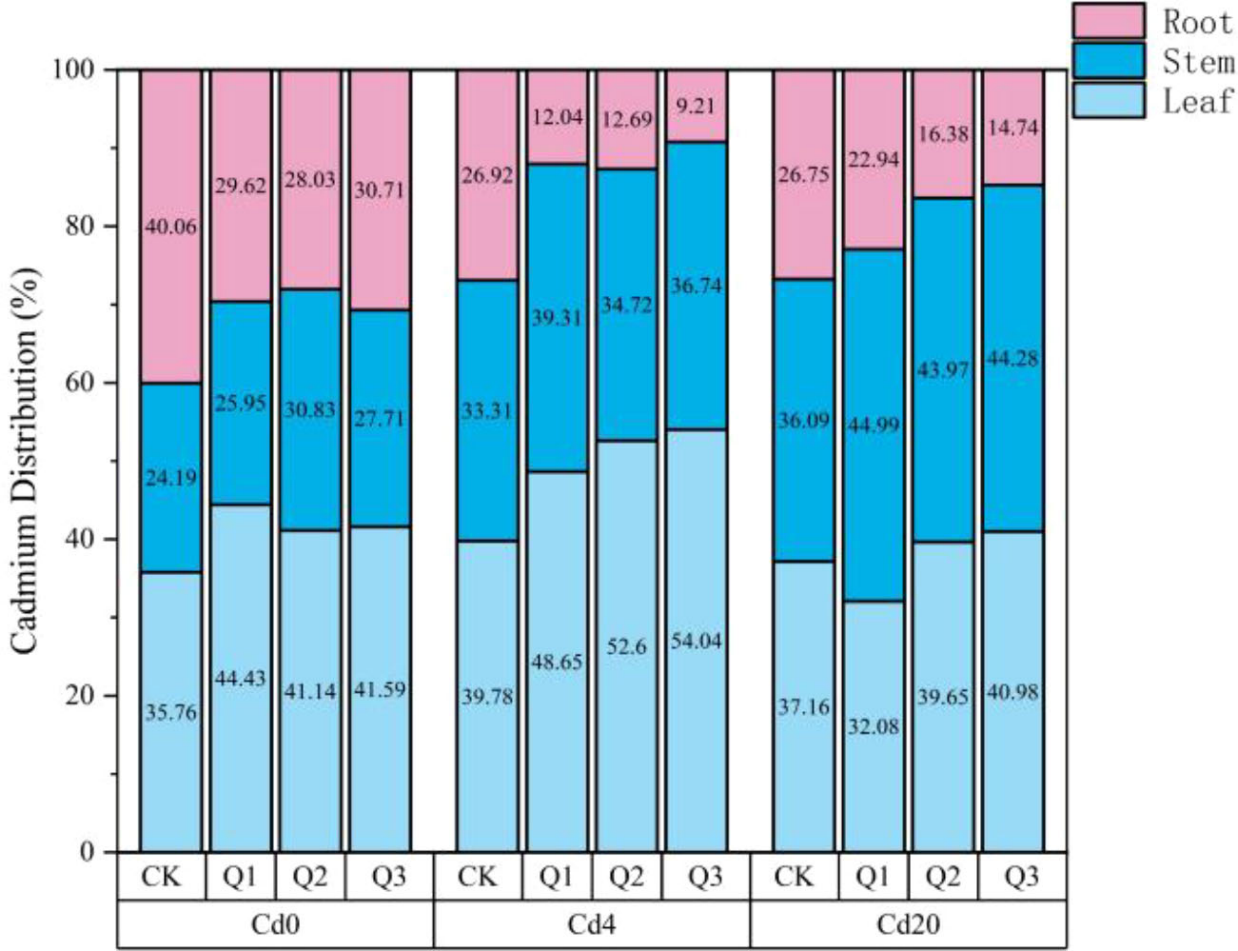
Comparison of Cd accumulation capacity in different organs of amaranth. The experiment included three Cd treatment levels (Cd0 = 0 mg·kg^-1^, Cd4 = 4 mg·kg^-1^, Cd20 = 20 mg·kg^-1^) and four quercetin application concentrations (CK = 0 mg·L^-1^, Q1 = 5 mg·L^-1^, Q2 = 10 mg·L^-1^, Q3 = 20 mg·L^-1^).

As shown in **Figure 3**, Cd concentrations in roots, stems, and leaves increased significantly with rising soil Cd levels, and exogenous quercetin further enhanced Cd accumulation capacity. Under Cd4 conditions, Q1 exerted the strongest promotive effect, elevating Cd content in roots, stems, and leaves by 54.37%, 253.28%, and 224.77%, respectively. Under Cd20 conditions, Q2 produced the greatest enhancement, with corresponding increases of 85.60%, 384.45%, and 294.97%, demonstrating that quercetin stimulates Cd uptake in a concentration dependent manner. As illustrated in **Figure 4**, Cd distribution among plant organs varied markedly across Cd stress levels and quercetin treatments, with an overall pattern of leaves > roots > stems, indicating that amaranth possesses strong aboveground translocation and accumulation capacity. In the absence of Cd, the majority of Cd was distributed in leaves (35.12%-44.01%), followed by roots (27.44%-38.82%) and stems (26.06%-28.55%). Under Cd4 stress, Cd allocation to aboveground tissues increased substantially: leaf Cd proportion rose from 39.55% in CK to 53.31% in Q3, while the proportion in roots declined from 26.87% to 9.15%, indicating that quercetin markedly promoted Cd transport from root to shoot, with Q3 exhibiting the strongest effect. Under Cd20 stress, Cd partitioning underwent further adjustment. Aboveground Cd allocation increased significantly across treatments, with Q3 achieving the highest proportion (85.26%), approximately 12.01% higher than CK (73.25%, *p* < 0.05), whereas the root proportion decreased from 27.22% to 14.88%. These findings collectively demonstrate that quercetin enhances both Cd uptake and translocation efficiency in amaranth, particularly under Cd20 exposure.

#### 3.2.2 Aboveground cadmium accumulation coefficient

As shown in **Table 4**, the Cd accumulation coefficient in the aboveground parts of amaranth under different Cd stress concentrations generally followed the pattern Cd4 > Cd20. Compared with the control group, the application of quercetin significantly increased the Cd accumulation coefficient of plants. With increasing quercetin concentration, the coefficient first increased and then decreased, reaching its maximum under the Q1 treatment condition at Cd4 and the Q2 treatment condition at Cd20. The maximum Cd accumulation coefficients in amaranth under different Cd stresses were 8.82 and 7.02, respectively, representing 2.47-fold and 3.44-fold increases compared to the control group. This indicates that quercetin most strongly promotes Cd accumulation in amaranth under Cd20 stress.

**Table 4:** Changes in above-ground enrichment coefficients under different treatment conditions.

C_Exogenous Cd_	CK	Q1	Q2	Q3
Cd4	2.54 ± 0.37b	8.82 ± 0.34a	8.66 ± 0.24a	8.34 ± 0.80a
Cd20	1.58 ± 0.06d	2.77 ± 0.14c	7.02 ± 0.30a	6.36 ± 0.23b

### 3.3 Effects of exogenous quercetin on stress resistance-related indices of *Amaranthus hypochondriacus* L. under cadmium stress

#### 3.3.1 Effects on antioxidant substances and total antioxidant capacity

**Figure 5:**
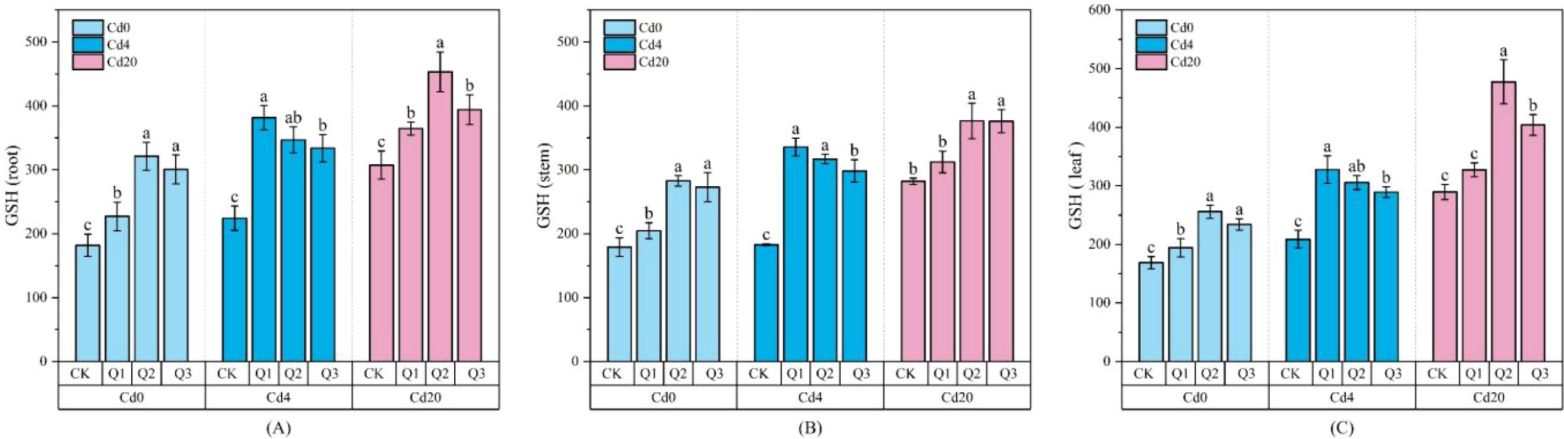
Changes in GSH content (μg·g^-1^ FW) under different treatment conditions: **(A)** root GSH content; **(B)** stem GSH content; **(C)** leaf GSH content. Values are expressed as mean ± SD (n=3). Bars with different letters differed significantly (*p* < 0.05) among the experimental groups. The experiment included three Cd treatment levels (Cd0 = 0 mg·kg^-1^, Cd4 = 4 mg·kg^-1^, Cd20 = 20 mg·kg^-1^) and four quercetin application concentrations (CK = 0 mg·L^-1^, Q1 = 5 mg·L^-1^, Q2 = 10 mg·L^-1^, Q3 = 20 mg·L^-1^).

**Figure 6:**
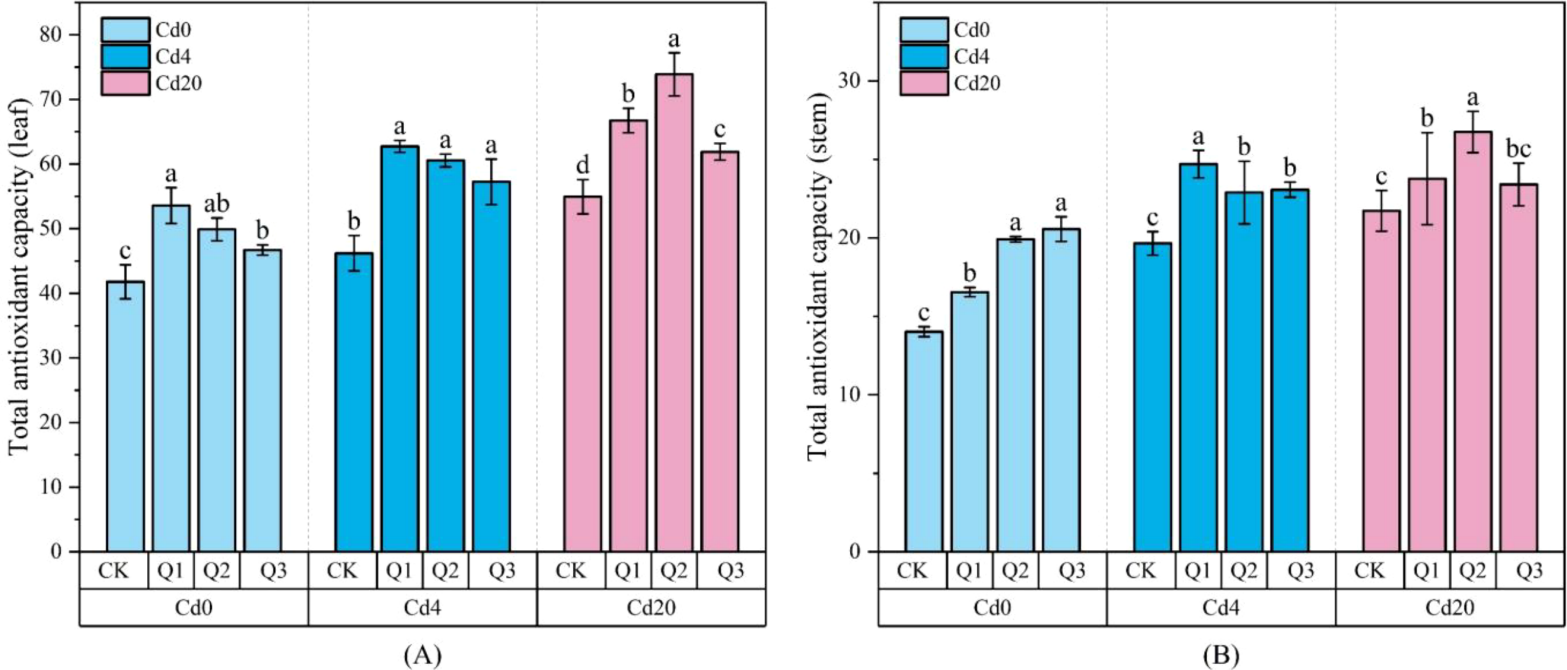
Changes in TAOC under different treatment conditions (μg·g^-1^ FW): **(A)** Total antioxidant capacity of leaf; **(B)** Total antioxidant capacity of stem. Values are expressed as mean ± SD (n=3). Bars with different letters differed significantly (*p* < 0.05) among the experimental groups. The experiment included three Cd treatment levels (Cd0 = 0 mg·kg^-1^, Cd4 = 4 mg·kg^-1^, Cd20 = 20 mg·kg^-1^) and four quercetin application concentrations (CK = 0 mg·L^-1^, Q1 = 5 mg·L^-1^, Q2 = 10 mg·L^-1^, Q3 = 20 mg·L^-1^).

As shown in **Figure 5**, Cd stress markedly induced GSH accumulation, with GSH levels in all organs increasing in parallel with Cd concentration, indicating activation of non-enzymatic antioxidant defenses in response to Cd toxicity. Following quercetin application, leaf GSH content increased further, reaching 477.32 μg·g^-1^ in the Cd20Q2 treatment, representing a 65% increase (*p* < 0.05) compared with the Cd20CK group. This suggests that quercetin may enhance the GSH-AsA cycle by activating GSH biosynthesis or promoting GSH regeneration, thereby strengthening ROS detoxification under Cd stress. In **Figure 6**, TAOC, a comprehensive indicator of ROS neutralization and redox maintenance, exhibited trends largely consistent with GSH dynamics, implying a synergistic enhancement of antioxidant performance by quercetin. TAOC values increased with rising Cd levels across treatments, and under Cd0 conditions, Q2 and Q3 displayed significant dose dependent increases relative to the control (*p* < 0.05), indicating that quercetin stimulates antioxidant defenses even in the absence of stress. Among different organs, leaves showed the greatest sensitivity to quercetin; TAOC increased by 42.90% under Cd4Q2 and by 35.30% under Cd20Q3 compared with their respective controls. The continuous rise in TAOC with increasing Cd levels further suggests that plants intensify antioxidant responses under severe stress, and that quercetin markedly amplifies this response through both direct radical scavenging activity and the induction of enzymatic and non-enzymatic antioxidant pathways. Together with GSH data, these findings demonstrate that quercetin enhances antioxidative capacity through multiple coordinated mechanisms, substantially improving plant resilience against Cd induced oxidative damage.

#### 3.3.2 Effect of exogenous quercetin on electrolyte leakage

**Figure 7:**
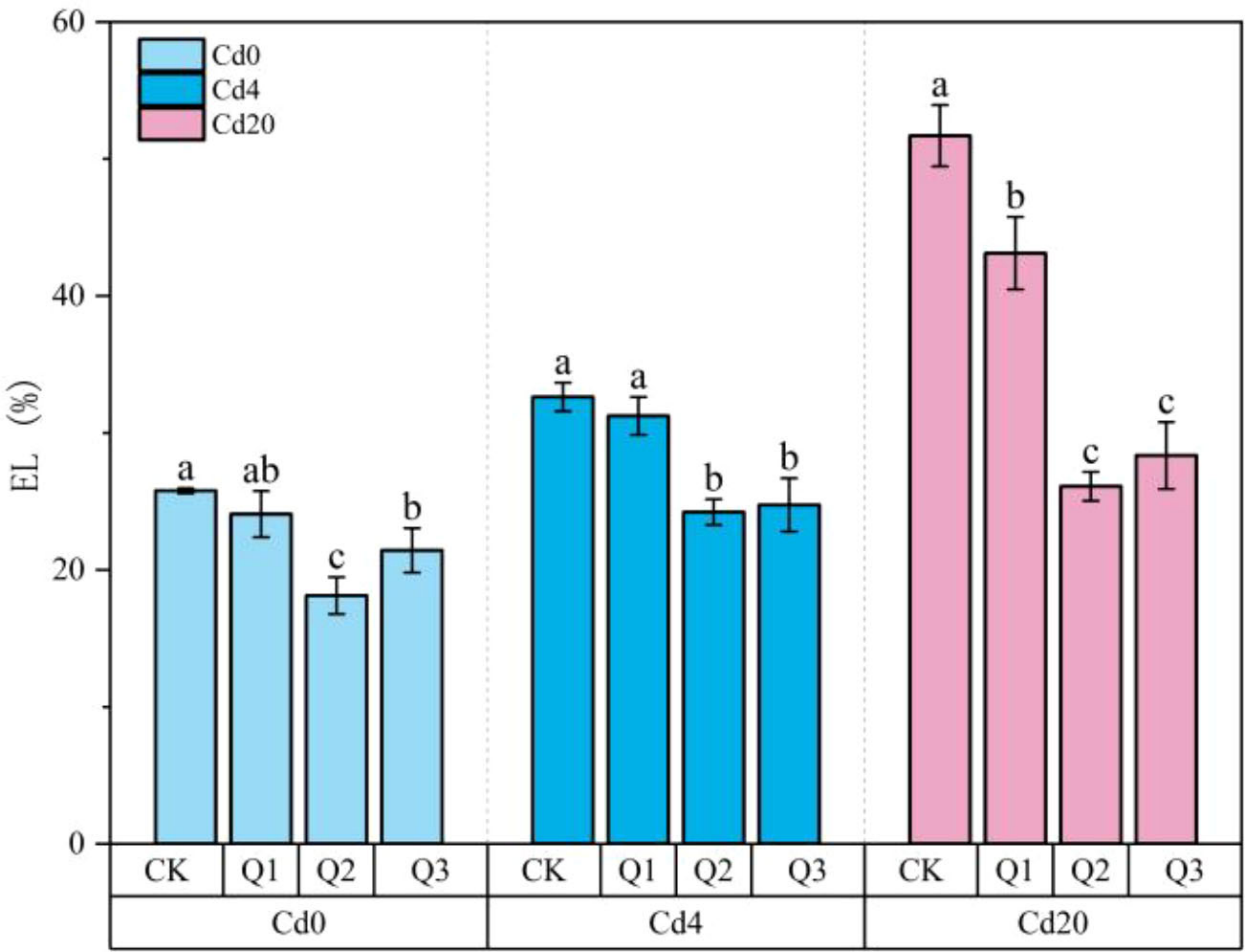
Changes in EL under different treatment conditions. Values are expressed as mean ± SD (n=3). Bars with different letters differed significantly (*p* < 0.05) among the experimental groups. The experiment included three Cd treatment levels (Cd0 = 0 mg·kg^-1^, Cd4 = 4 mg·kg^-1^, Cd20 = 20 mg·kg^-1^) and four quercetin application concentrations (CK = 0 mg·L^-1^, Q1 = 5 mg·L^-1^, Q2 = 10 mg·L^-1^, Q3 = 20 mg·L^-1^).

EL is a key indicator of cell membrane integrity and an important measure of plant stress resistance. As shown in **Figure 7**, both Cd4 and Cd20 treatments exhibited higher EL than the Cd0 group, confirming that Cd induced oxidative stress compromises membrane stability in a concentration-dependent manner. In the absence of quercetin, EL reached 32.63% and 51.7% in the Cd4 and Cd20 groups, respectively. Following quercetin application, EL declined across all Cd treatments, indicating that quercetin significantly alleviated membrane damage (*p* < 0.05). Under Q2 treatment, EL decreased to 24.22% in Cd4 plants and to 26.11% in Cd20 plants, corresponding to reductions of 25.78% and 49.50%, respectively. Even under Cd0, quercetin reduced EL by approximately 50% compared with the control (*p* < 0.05), suggesting that quercetin can stabilize membrane structure or enhance basal antioxidant defenses independent of Cd exposure. The minimum EL values achieved under different Cd treatments did not differ significantly, implying the presence of a saturation threshold in quercetin mediated membrane protection. This pattern is consistent with the observed increases in GSH and TAOC.

#### 3.3.3 Effects on phenylalanine and phenolic acids

As shown in **Tables 5**–**7**, exogenous quercetin markedly increased the levels of phenylalanine, cinnamic acid, and p-coumaric acid in amaranth. Under Cd4 stress, the content of p-coumaric acid in the Q3 treatment reached 1.41, 0.95, and 3.82 μmol·g^-1^ in roots, stems, and leaves, respectively, representing increases of 58.43%, 46.15%, and 37.91% compared with the CK group (0.89, 0.65, and 2.77 μmol·g^-1^ FW). A similar pattern was observed under Cd20 stress, where Q3 again yielded the highest values (1.52, 0.92, and 3.87 μmol·g^-1^ FW). Phenylalanine and cinnamic acid showed the same dose-dependent response, increasing progressively with quercetin application and reaching their maxima under the Q3 treatment. These results indicate that quercetin activates the phenylpropanoid pathway, thereby enhancing the production of key upstream precursors which can subsequently support elevated flavonoid biosynthesis. This metabolic activation may contribute to improved antioxidant capacity and enhanced tolerance to Cd stress.

**Table 5:** Changes in phenylalanine under different treatment conditions (μmol·g^-1^ DW).

C_Exogenous Cd_	C_Exogenous Quercetin_	Phenylalanine content (μmol·g^-1^ DW)		
		Root	Stem	Leaf
Cd0	CK	16.44 ± 1.11d	14.36 ± 1.20d	22.16 ± 0.67c
Cd0	Q1	19.17 ± 0.57c	18.66 ± 1.20c	26.26 ± 2.38c
Cd0	Q2	22.74 ± 0.76b	22.92 ± 0.34b	31.59 ± 1.69b
Cd0	Q3	32.25 ± 0.59a	28.55 ± 0.74a	42.71 ± 3.89a
Cd4	CK	21.50 ± 1.67c	24.94 ± 1.33b	33.03 ± 1.15c
Cd4	Q1	26.20 ± 1.40b	19.80 ± 2.42c	37.03 ± 2.55b
Cd4	Q2	31.78 ± 0.52a	20.34 ± 1.59c	34.36 ± 1.56c
Cd4	Q3	32.49 ± 1.88a	28.38 ± 0.98a	46.16 ± 2.61a
Cd20	CK	26.23 ± 2.10c	23.88 ± 0.79c	37.43 ± 2.35c
Cd20	Q1	30.61 ± 1.06b	26.24 ± 0.62b	44.18 ± 3.41b
Cd20	Q2	31.81 ± 2.33b	26.90 ± 0.58b	40.88 ± 3.56a
Cd20	Q3	35.43 ± 2.78a	28.83 ± 1.26a	45.63 ± 3.26a

**Table 6:** Changes in cinnamic acid under different treatment conditions (μmol·g^-1^ FW).

C_Exogenous Cd_	C_Exogenous Quercetin_	Cinnamic acid content (μmol·g^-1^ FW)		
		Root	Stem	Leaf
Cd0	CK	0.51 ± 0.01b	0.38 ± 0.02c	1.32 ± 0.05c
Cd0	Q1	0.67 ± 0.02b	0.42 ± 0.01c	1.53 ± 0.07b
Cd0	Q2	0.72 ± 0.08b	0.57 ± 0.05b	2.04 ± 0.09a
Cd0	Q3	1.33 ± 0.57a	0.73 ± 0.03a	1.91 ± 0.09a
Cd4	CK	0.76 ± 0.03b	0.48 ± 0.03d	1.7 ± 0.03c
Cd4	Q1	0.82 ± 0.05b	0.53 ± 0.02c	2.02 ± 0.08b
Cd4	Q2	0.84 ± 0.07b	0.59 ± 0.03b	2.15 ± 0.11b
Cd4	Q3	1.20 ± 0.03a	0.67 ± 0.04a	2.83 ± 0.24a
Cd20	CK	0.87 ± 0.04c	0.57 ± 0.02c	2.04 ± 0.07c
Cd20	Q1	1.02 ± 0.05b	0.63 ± 0.02b	2.33 ± 0.18b
Cd20	Q2	0.97 ± 0.06b	0.66 ± 0.05b	2.36 ± 0.17b
Cd20	Q3	1.06 ± 0.03a	0.75 ± 0.03a	2.59 ± 0.04a

**Table 7:** Changes in p-coumaric acid under different treatment conditions (μmol·g^-1^ FW).

C_Exogenous Cd_	C_Exogenous Quercetin_	p-Coumaric acid content (μmol·g^-1^ FW)		
		Root	Stem	Leaf
Cd0	CK	0.73 ± 0.03d	0.46 ± 0.02d	1.96 ± 0.07c
Cd0	Q1	0.83 ± 0.06c	0.54 ± 0.06c	2.49 ± 0.08b
Cd0	Q2	1.13 ± 0.05b	0.65 ± 0.02b	2.7 ± 0.14b
Cd0	Q3	1.40 ± 0.06a	0.85 ± 0.06a	3.51 ± 0.21a
Cd4	CK	0.89 ± 0.08c	0.65 ± 0.03c	2.74 ± 0.26c
Cd4	Q1	1.18 ± 0.06b	0.67 ± 0.04c	3.43 ± 0.21b
Cd4	Q2	1.27 ± 0.05b	0.79 ± 0.03b	2.95 ± 0.02c
Cd4	Q3	1.41 ± 0.10a	0.95 ± 0.03a	3.82 ± 0.09a
Cd20	CK	1.23 ± 0.08b	0.83 ± 0.06b	3.48 ± 0.02b
Cd20	Q1	1.28 ± 0.06b	0.83 ± 0.02b	3.64 ± 0.09b
Cd20	Q2	1.38 ± 0.11b	0.9 ± 0.06b	3.37 ± 0.26b
Cd20	Q3	1.52 ± 0.10a	0.92 ± 0.02a	3.87 ± 0.27a

#### 3.3.4 Effects on flavonoid metabolism

**Figure 8:**
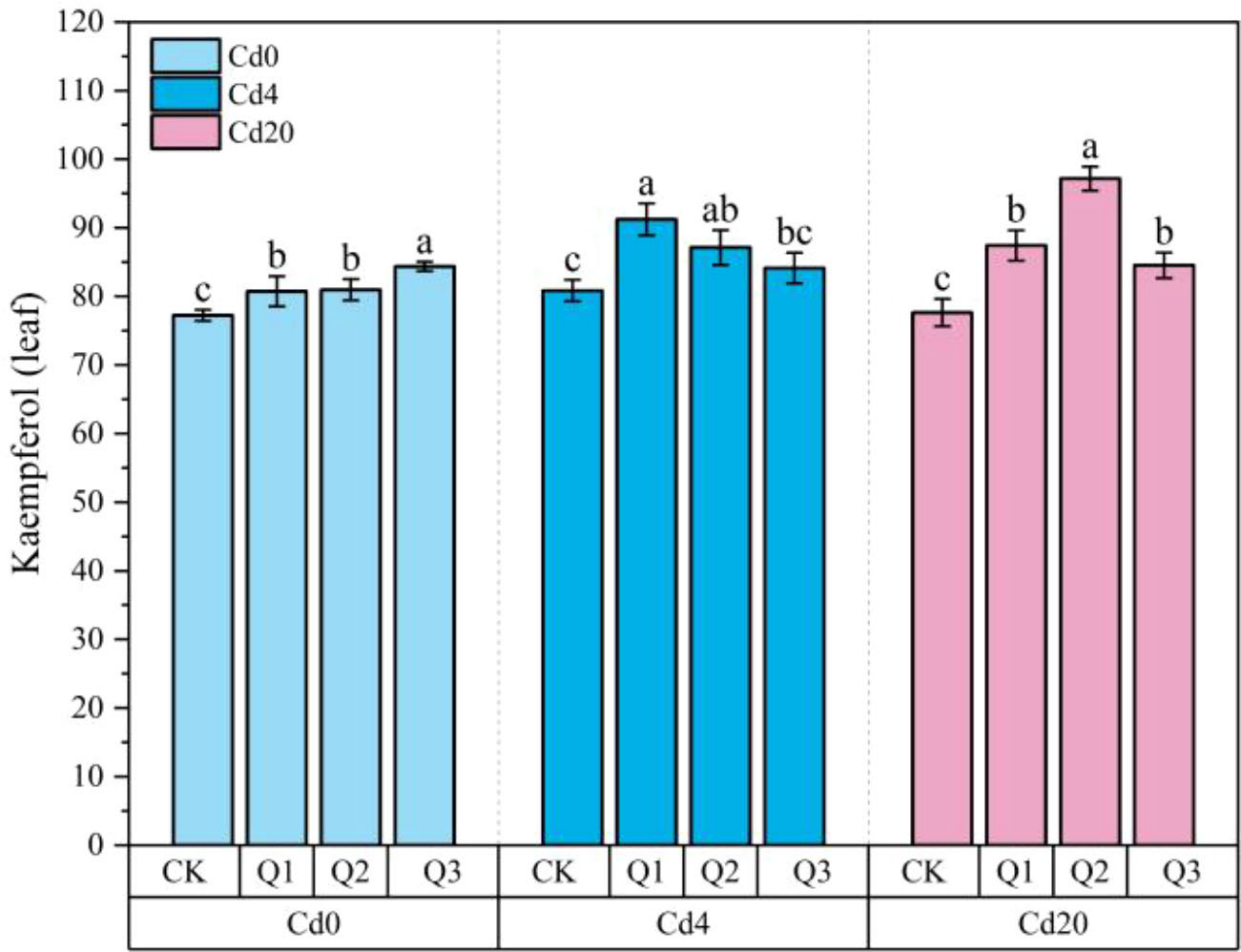
Changes in kaempferol content in leaves under different treatment conditions (μg·g^-1^ FW). Values are expressed as mean ± SD (n=3). Bars with different letters differed significantly (*p* < 0.05) among the experimental groups. The experiment included three Cd treatment levels (Cd0 = 0 mg·kg^-1^, Cd4 = 4 mg·kg^-1^, Cd20 = 20 mg·kg^-1^) and four quercetin application concentrations (CK = 0 mg·L^-1^, Q1 = 5 mg·L^-1^, Q2 = 10 mg·L^-1^, Q3 = 20 mg·L^-1^).

**Figure 9:**
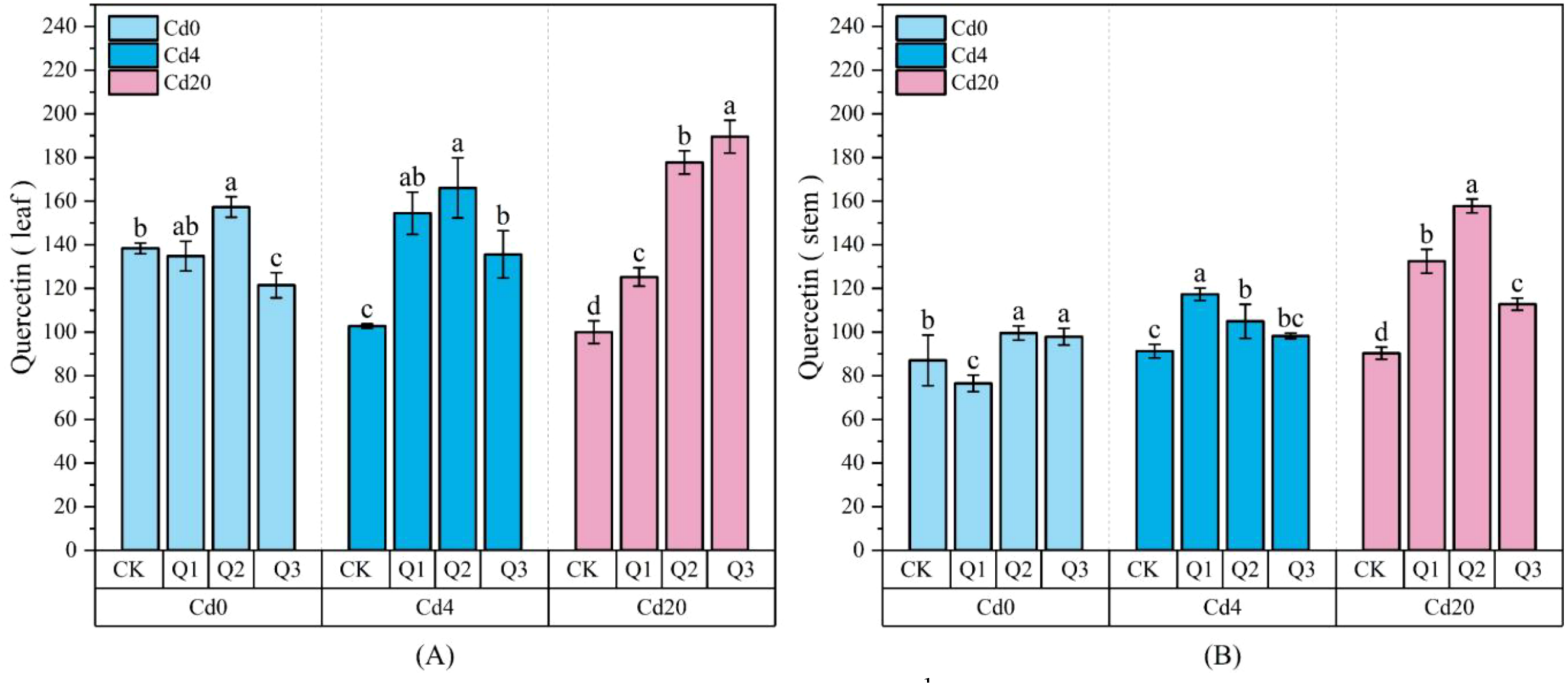
Changes in stem and leaf quercetin content (μg·g^-1^ FW) under different treatment conditions **(A)** Leaf quercetin content; **(B)** Stem quercetin content. Values are expressed as mean ± SD (n=3). Bars with different letters differed significantly (*p* < 0.05) among the experimental groups. The experiment included three Cd treatment levels (Cd0 = 0 mg·kg^-1^, Cd4 = 4 mg·kg^-1^, Cd20 = 20 mg·kg^-1^) and four quercetin application concentrations (CK = 0 mg·L^-1^, Q1 = 5 mg·L^-1^, Q2 = 10 mg·L^-1^, Q3 = 20 mg·L^-1^).

**Figure 10:**
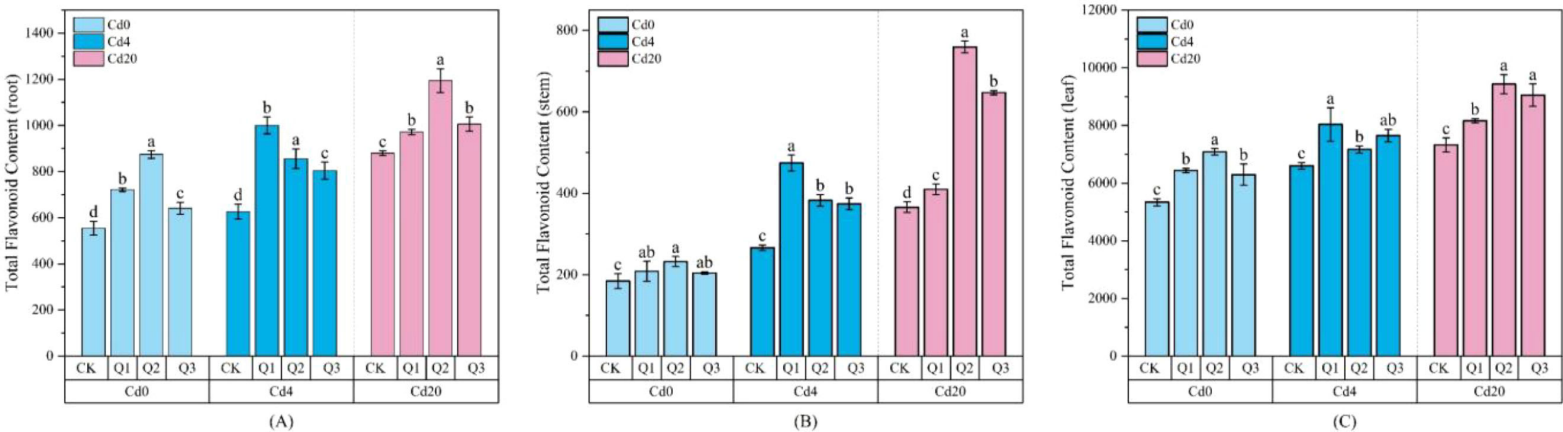
Changes in total flavonoids under different treatment conditions (μg·g^-1^ FW): **(A)** Total flavonoid content in roots; **(B)** Total flavonoid content in stems; **(C)** Total flavonoid content in leaves. Values are expressed as mean ± SD (n=3). Bars with different letters differed significantly (*p* < 0.05) among the experimental groups. The experiment included three Cd treatment levels (Cd0 = 0 mg·kg^-1^, Cd4 = 4 mg·kg^-1^, Cd20 = 20 mg·kg^-1^) and four quercetin application concentrations (CK = 0 mg·L^-1^, Q1 = 5 mg·L^-1^, Q2 = 10 mg·L^-1^, Q3 = 20 mg·L^-1^).

In our testing of flavonoids across roots, stems, and leaves, kaempferol and quercetin were nearly undetectable in root samples, and kaempferol was also absent in stems. **Figure 8** illustrates the changes in leaf kaempferol content, while **Figures 9** and **10** depict the effects of quercetin treatments on quercetin levels in stems and leaves, as well as total flavonoid content in all organs of amaranth. Under Cd stress conditions, kaempferol content increased in response to quercetin application, reaching a maximum of 87.13 μg·g^-1^ in the Cd4Q2 treatment. In both Cd4 and Cd20 groups, exogenous quercetin significantly enhanced the accumulation of quercetin and total flavonoids. Under Cd20 stress, leaf quercetin content in the Q2 and Q3 treatments reached 177.71 μg·g^-1^ and 189.53 μg·g^-1^, representing 77.76% and 89.89% increases, respectively, compared with the Cd20CK group (99.97 μg·g^-1^). Total flavonoid content displayed a similar pattern: under the Cd20Q2 treatment, total flavonoid levels in roots, stems, and leaves reached 1194.35, 759.52, and 9435.05 μg·g^−1^, respectively, corresponding to increases of approximately 28.80%, 107.76%, and 35.80% relative to the control, with the most pronounced relative increase observed in stems. The stimulation of flavonoid biosynthesis by quercetin is likely attributable to its structural properties as a flavonoid molecule, which may enhance precursor availability, such as phenylalanine, cinnamic acid, and p-coumaric acid, through activation of the phenylpropanoid pathway. This metabolic activation ultimately promotes greater flavonoid accumulation, strengthening antioxidant capacity and improving heavy metal detoxification.

Under Cd20 stress, the promotive effect of quercetin on flavonoid metabolism was particularly pronounced, suggesting that its contribution to stress resistance extends beyond mitigating oxidative damage. By enhancing flavonoid production, quercetin may also facilitate the immobilization and sequestration of Cd within plant tissues.

### 3.4 Combined effects of cadmium stress and quercetin application on physiological responses of plants

**Figure 11:**
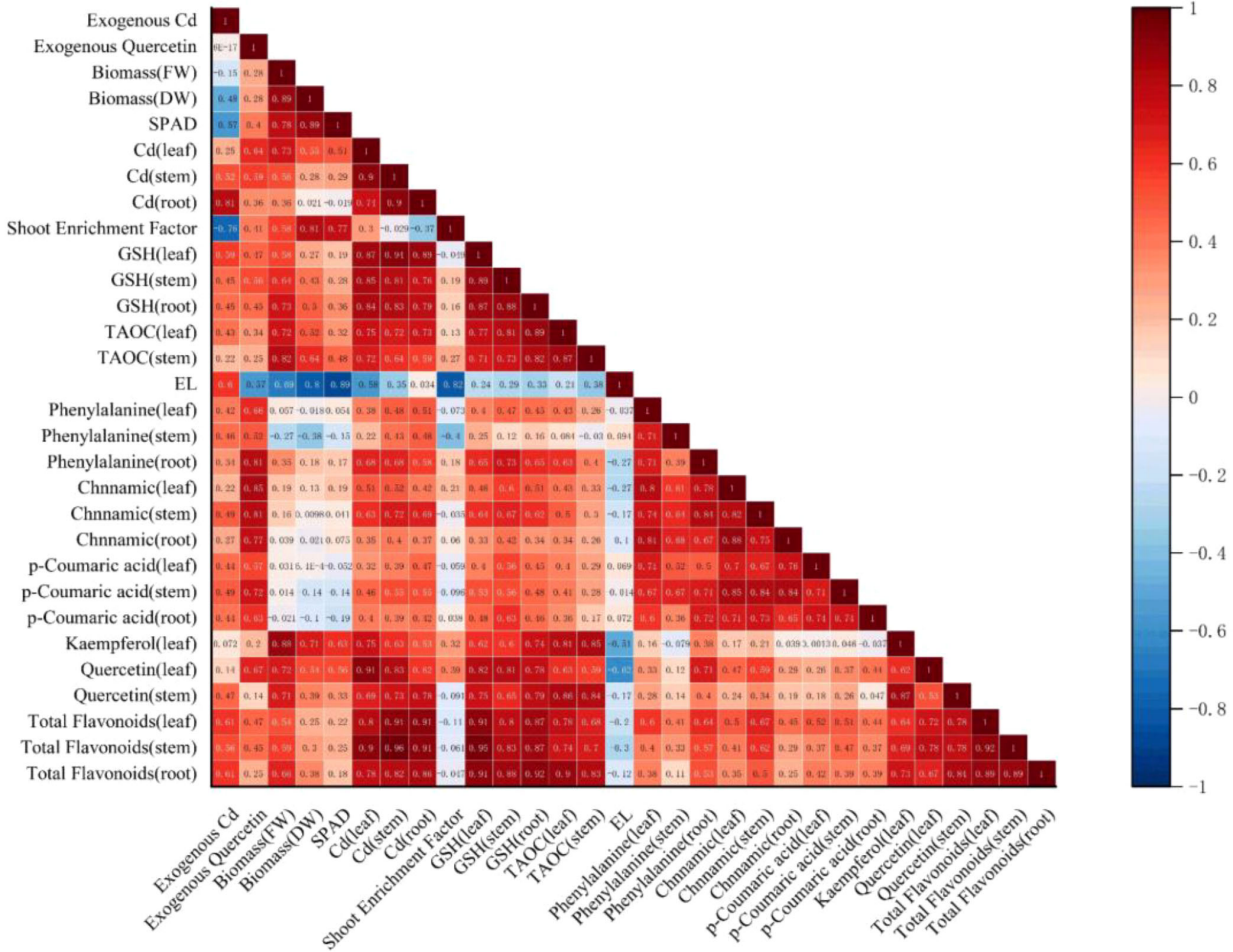
Correlation heatmap of various indicators. It ranges from -1 to 1, whereby -1 means a perfect negative linear relationship between variables, 1 indicates a perfect positive linear relationship between variables and 0 indicates that there is no relationship between studied variables. Significant level (*p* < 0.05).

Correlation and linear regression analyses were conducted using Origin 2024 and SPSS 27. As shown in **Figure 11**, the correlation heatmap illustrates the relationships among key physiological and metabolic indicators. Pearson correlation analysis was employed to clarify how Cd stress and quercetin application jointly influence plant physiological responses. The results revealed a significant positive correlation (*p* < 0.05) between Cd stress intensity and the accumulation of phenolic acids and flavonoids. Endogenous quercetin content increased in parallel with exogenous quercetin application, with a correlation coefficient of 0.67. GSH and TAOC also showed strong positive correlations with quercetin concentration (*p* < 0.05), indicating activation of the GSH-AsA cycle. Consistent with these physiological enhancements, Cd accumulation capacity was also significantly increased (*p* < 0.05, *r* = 0.41), further supporting the conclusion that quercetin strengthens the phytoremediation potential of amaranth through coordinated metabolic and antioxidant regulation. In contrast, EL also exhibited significant negative correlations with multiple metabolic indicators.

This study demonstrates that exogenous quercetin mitigates Cd stress in amaranth through coordinated metabolic reprogramming, as reflected by strong correlations between quercetin concentration and key metabolites, antioxidant indicators, and Cd accumulation traits. Overall, quercetin application showed significant positive correlations with phenylpropanoid precursors (phenylalanine, cinnamic acid, and p-coumaric acid), major flavonoid metabolites (quercetin, kaempferol, and total flavonoids), antioxidant markers (GSH and TAOC), and aboveground Cd accumulation, while exhibiting a significant negative correlation with EL. These relationships form the metabolic basis by which quercetin enhances Cd tolerance and phytoextraction efficiency, consistent with its dual regulatory roles in the phenylpropanoid flavonoid and GSH-PCs detoxification pathways.

## 4 Discussion

### 4.1 Effects of quercetin on cadmium accumulation in roots, stems and leaves and growth inhibition of *Amaranthus hypochondriacus* L. under cadmium stress

Cd stress typically exerts a significant inhibitory effect on plant growth. Exogenous quercetin not only regulates Cd accumulation and distribution patterns in the roots, stems, and leaves of amaranth, but also effectively alleviates growth inhibition induced by cadmium stress. Exogenous quercetin significantly increased Cd content in the roots, stems, and leaves of amaranth, with the most pronounced effect observed in the Cd20Q2 treatment. Furthermore, quercetin markedly enhanced the translocation of Cd from roots to the aboveground tissues (Aslam et al., 2022). Under Cd20, the proportion of Cd allocated to leaves increased from 35.12% in the control group to 53.31% in the Q2 group, and no significant growth inhibition was observed in the plants. The most important finding of this study is that quercetin simultaneously enhances both Cd tolerance and accumulation in amaranth.

These results are consistent with those reported by Li et al. (2022); Singh et al. (2021) and Yang et al. (2022), indicating that flavonoids can enhance Cd accumulation in plants via chelation and detoxification. Different from the conventional strategy that mostly applies quercetin to reduce heavy metal accumulation in crops, the present study simultaneously improved Cd tolerance and Cd enrichment capacity in hyperaccumulator plants, thereby providing a novel and feasible approach for enhancing the efficiency of phytoremediation. However, the present findings differ markedly from those reported in leafy vegetables such as spinach and lettuce. Li et al. (2024); Tang et al. (2023) and Seyfferth et al. (2024) demonstrated that exogenous quercetin in edible crops primarily alleviates heavy metal accumulation by suppressing Cd uptake in roots and restricting its translocation to edible aboveground tissues. Conversely, this study, focused on phytoremediation, significantly enhanced Cd absorption and translocation in amaranth purpureus. These divergent regulatory mechanisms and distinct application objectives underscore the context specific of quercetin function in plants.

### 4.2 Regulatory effects of quercetin on the antioxidant system of *Amaranthus hypochondriacus* L. under cadmium stress

**Figure 12:**
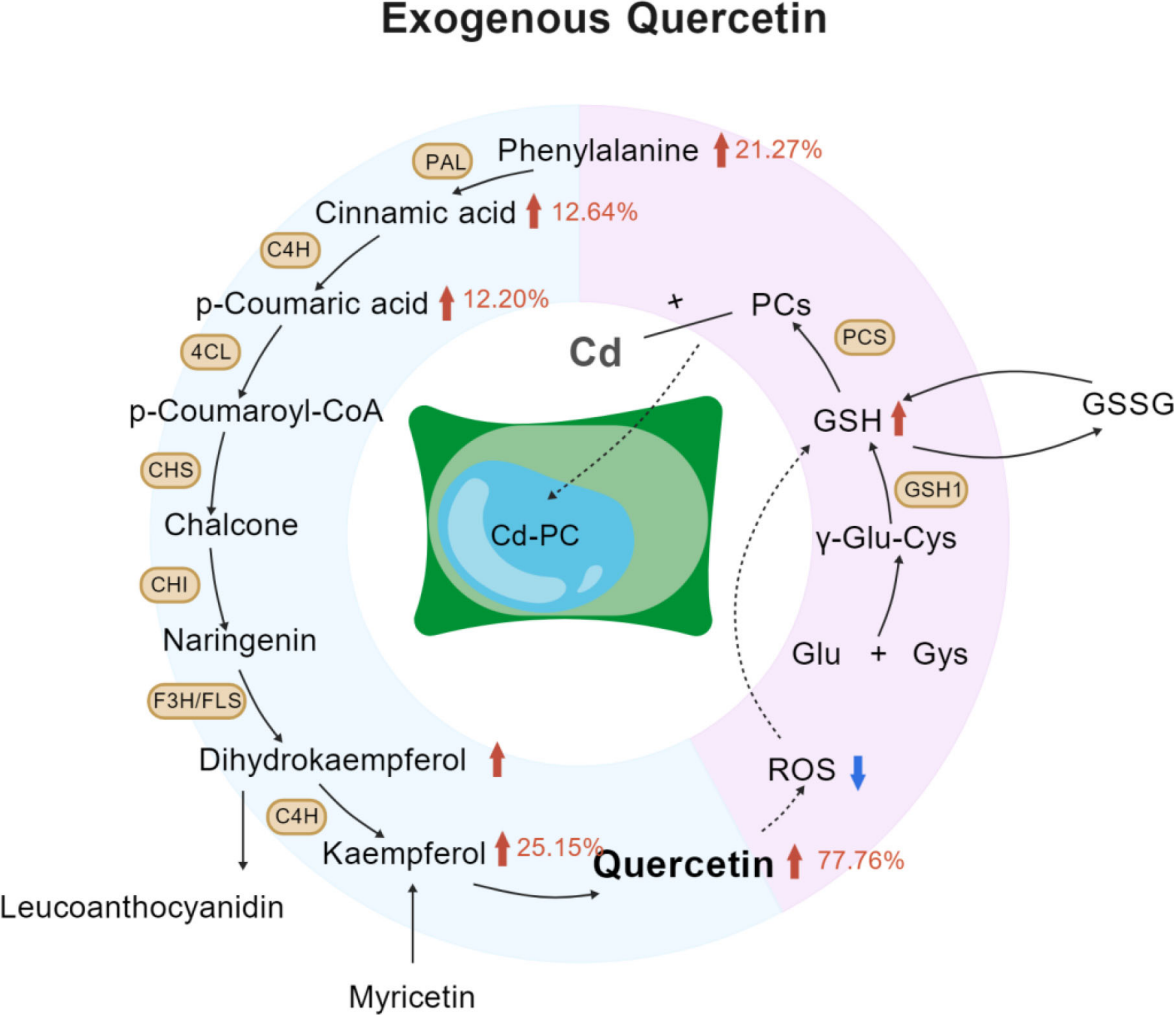
Schematic of the metabolic repair mechanism enhanced by exogenous quercetin.

Quercetin significantly activated the antioxidant system and maintained cell membrane stability, markedly elevated GSH and TAOC content, and decreased EL. EL was decreased by 49.50% under the Cd20Q2 treatment. These results collectively reveal a sequential cascade in which increased exogenous quercetin leads to elevated levels of GSH and flavonoids, which in turn reduces ROS accumulation and ultimately alleviates electrolyte leakage, thereby directly confirming the antioxidative protective role of quercetin. (shown in **Figure 12**) (Panche et al., 2021). This result is highly consistent with the findings of Arikan et al. (2023); Parvin et al. (2019) and Wang et al. (2025) further verifying that quercetin in promotes GSH biosynthesis and alleviates membrane lipid peroxidation. This study demonstrated that exogenous quercetin and the endogenous GSH and quercetin system can exert a synergistic antioxidant effect, thereby enhancing the stability and efficiency of the overall defense system. When exogenous quercetin reached Q3, the growth performance, quercetin metabolism, antioxidant system, and Cd accumulation capacity of amaranth all decreased significantly, showing a typical hormetic effect (Aslam et al., 2022). From the perspective of oxidative stress, excessive quercetin can be converted into pro-oxidants via autoxidation, directly triggering massive ROS accumulation in plants, aggravating membrane lipid peroxidation, and ultimately impairing cell membrane structural stability. This mechanism is consistent with the findings of Aslam et al. (2022); Carrillo-Martinez et al. (2024); Jia et al. (2010), and Robaszkiewicz et al. (2007) who reported that high concentrations of quercetin may exert pro-oxidant effects under specific conditions.

### 4.3 Synergistic regulatory mechanisms of quercetin on physiological and metabolic responses of *Amaranthus hypochondriacus* L. under cadmium stress

Exogenous quercetin regulates the phenylpropanoid flavonoid metabolic pathway in amaranth under Cd stress in a concentration dependent manner (Liu et al., 2025). Treatment with appropriate concentrations of quercetin significantly elevated the contents of upstream precursors, including phenylalanine, cinnamic acid, and p-coumaric acid. Meanwhile, it also promoted the accumulation of downstream products, such as endogenous quercetin, silybin, and total flavonoids. In the optimal treatment group (Cd20Q2), the contents of endogenous quercetin and kaempferol were significantly increased by 77.76% and 25.15%, respectively, compared with the control treatment. This comprehensive synergistic upregulation suggests that quercetin activates the entire metabolic pathway, rather than merely acting on individual metabolites (Han et al., 2025). This result is consistent with the findings reported by Jańczak-Pieniążek et al. (2022a); Migut et al. (2022) and Zhang and Xia (2025), confirming that exogenous quercetin activates the phenylpropanoid pathway and promotes endogenous quercetin biosynthesis, thereby improving ROS scavenging and heavy metal chelation capabilities.

However, phenylpropanoid flavonoid metabolic pathway analysis further revealed that excessive quercetin application exerted an inhibitory effect. At high quercetin concentrations (Q3), a downstream negative feedback regulation was triggered, as indicated by decreased endogenous quercetin and kaempferol contents accompanied by significant accumulation of upstream precursors, including phenylalanine, cinnamic acid, and p-coumaric acid. This metabolic pattern suggests that excessive quercetin restricts the conversion of precursors into downstream flavonoids, leading to metabolic flux obstruction and potential carbon and nitrogen imbalance. Such metabolic disruption was further aggravated by impaired photosynthesis, compromised membrane stability, and weakened antioxidant capacity, forming a vicious cycle of metabolic disorder that ultimately inhibited plant growth and reduced Cd accumulation efficiency. These observations are consistent with previous studies, verifying that excessive exogenous quercetin can induce metabolic negative feedback and disturb normal physiological metabolism in plants (Aslam et al., 2022; Akram et al., 2024; Han et al., 2025; Kang et al., 2022; Jańczak-Pieniążek et al., 2025).

Unlike in food crops such as rice and wheat, where quercetin induced pathway activation is frequently accompanied by growth inhibition, amaranth exhibited no obvious growth inhibition under quercetin treatment despite pronounced metabolic activation (Fedoreyeva et al., 2024; Jańczak-Pieniążek et al., 2022b; Xu et al., 2019). These findings highlight the unique physiological plasticity of amaranth as a Cd hyperaccumulator.

The regulatory effect of quercetin on amaranth under Cd stress is manifested not only in the overall activation of the phenylpropanoid flavonoid metabolic pathway but also in the induction of systemic responses across multiple physiological levels. Correlation analysis (**Figure 11**) revealed that exogenous quercetin displayed significant positive correlations with biomass, SPAD values, and Cd accumulation capacity, while exhibiting significant negative correlations with indices of membrane damage. This correlational profile further validates the dual protective and reparative roles of flavonoids under heavy metal stress (Aslam et al., 2022; Kang et al., 2022). Meanwhile, quercetin exhibited a strong correlation with key indices of the endogenous antioxidant system, suggesting that it synergistically alleviates oxidative damage by activating the antioxidant defense system. This is consistent with the flavonoid−mediated heavy metal detoxification mechanisms documented in previous studies (Parvin et al., 2019; Wang et al., 2025). From a holistic regulatory perspective, quercetin enhances Cd tolerance and restorative capacity in amaranth by orchestrating multiple interconnected physiological processes, including photosynthesis, antioxidant activity, and Cd accumulation.

Based on the above results, this study systematically elucidated the inhibitory mechanism of high concentration quercetin from two perspectives: oxidative stress induced damage and the negative feedback regulation of metabolic pathways. Moderate quercetin concentrations activate the phenylpropanoid flavonoid pathway and enhance antioxidant capacity, thereby improving Cd tolerance and accumulation, whereas excessive quercetin triggers metabolic negative feedback and physiological imbalance. This not only provides an important theoretical basis but also offers a concentration reference for the rational application of quercetin in the phytoremediation of Cd contaminated soils.

## 5 Conclusion

Exogenous quercetin significantly enhanced Cd tolerance and phytoextraction performance in amaranth. Among the treatments, the Cd20Q2 group showed the optimal performance, with improved plant growth, reduced membrane damage, and enhanced Cd enrichment. Mechanistically, quercetin stimulated the phenylpropanoid flavonoid pathway, promoting the accumulation of phenylalanine, phenolic acids, and endogenous flavonoids, and synergistically enhanced antioxidant capacity through the GSH-AsA cycle. However, when quercetin concentration reached Q3, negative feedback inhibition occurred, reducing some of the beneficial effects. In conclusion, quercetin enhances the phytoremediation efficiency of amaranth in Cd contaminated soils by regulating phenylpropanoid flavonoid metabolism, the antioxidant system, and Cd transport capacity. As an environmentally friendly and non-toxic compound, quercetin shows promising potential for the remediation of Cd contaminated soils.

## Data Availability

The original contributions presented in the study are included in the article/**Supplementary Material**. Further inquiries can be directed to the corresponding authors.
