# Supplementary material for: Study on the physiological and metabolic mechanisms of exogenous quercetin in cadmium hyperaccumulator Amaranthus hypochondriacus L
Source: Front Plant Sci. 2026 Apr 14;17:1788620. doi: 10.3389/fpls.2026.1788620 (PMC13122773; doi:10.3389/fpls.2026.1788620)
Supplement: Supplementary file 1 [file Supplementaryfile1.docx]

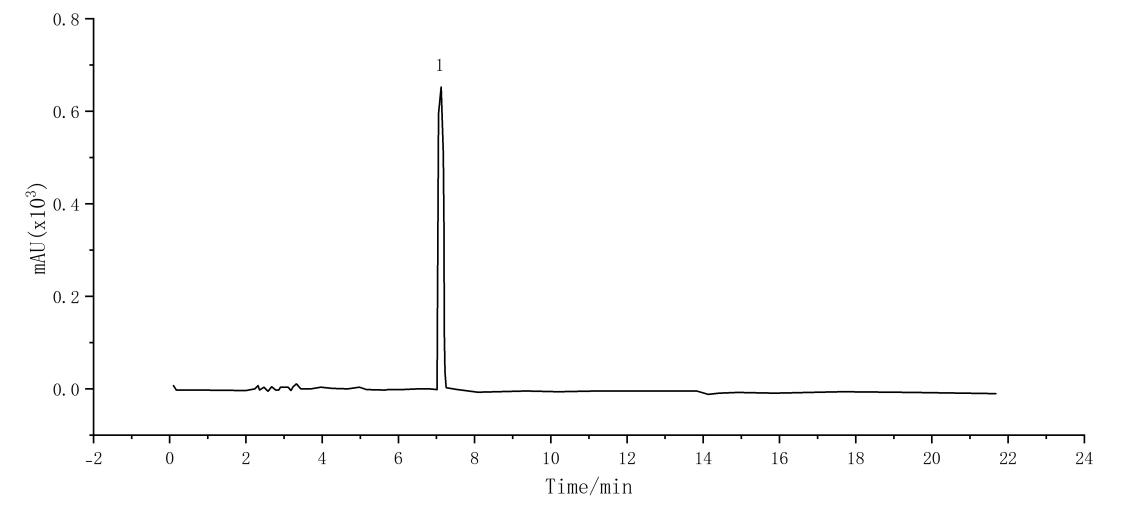


**
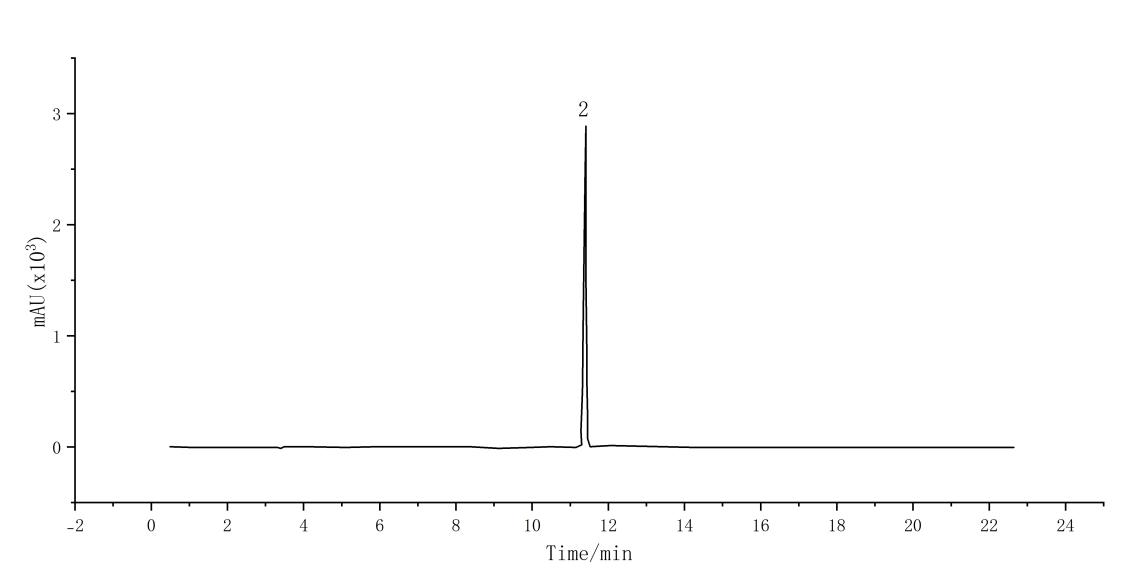
Figure 1.** HPLC chromatogram of L-phenylalanine standard


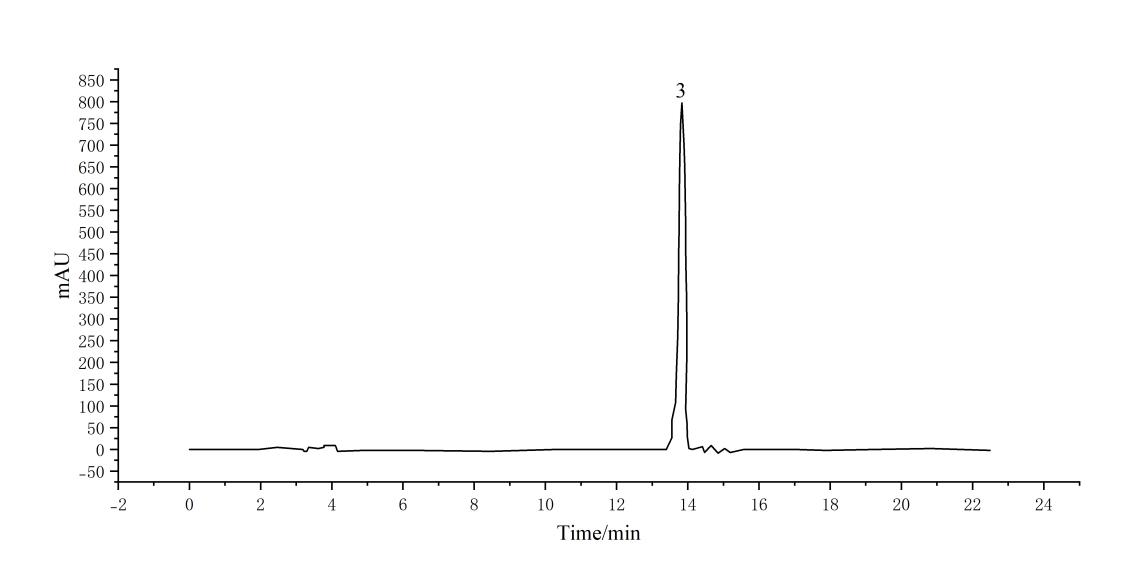
**Figure 2.** HPLC chromatogram of cinnamic acid standard

**Figure 3.** HPLC chromatogram of p-coumaric acid standard


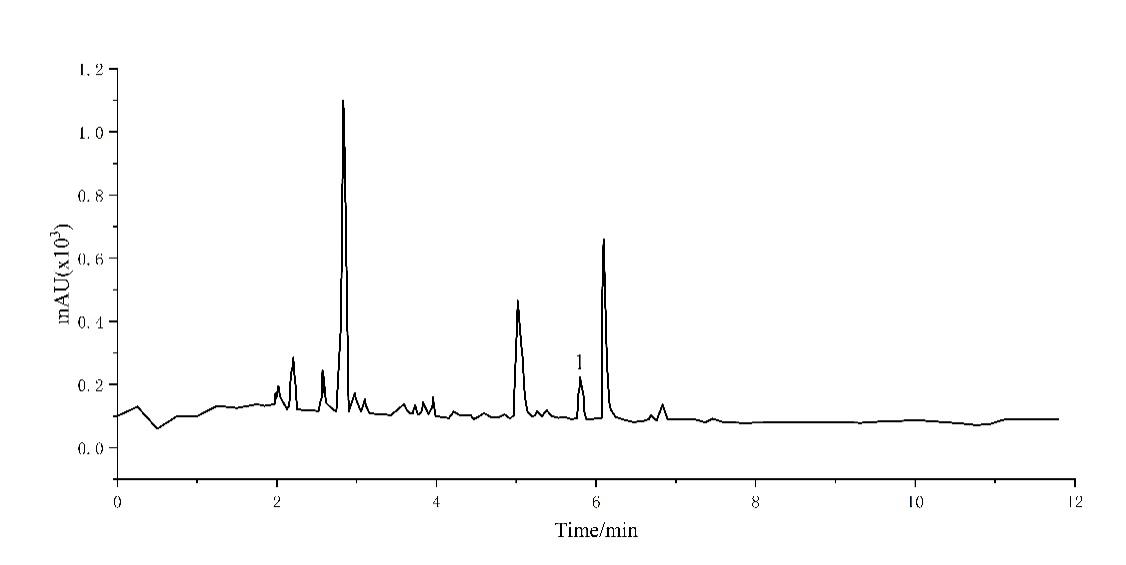


**Figure 4.** Chromatogram of phenylalanine in leaves under Cd20Q2 conditions


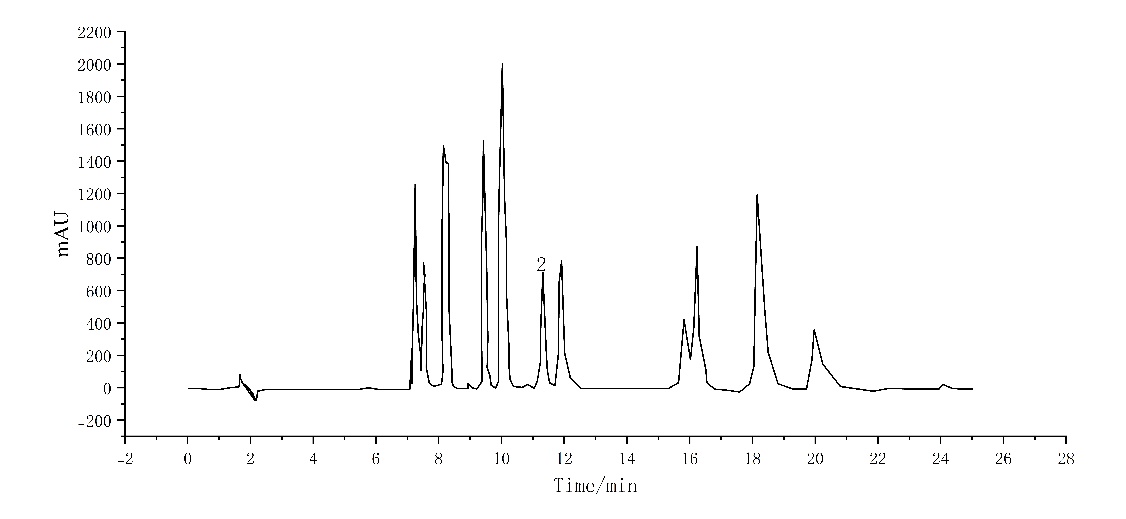


**Figure 5.** Chromatogram of cinnamic acid in leaves under Cd20Q2 conditions


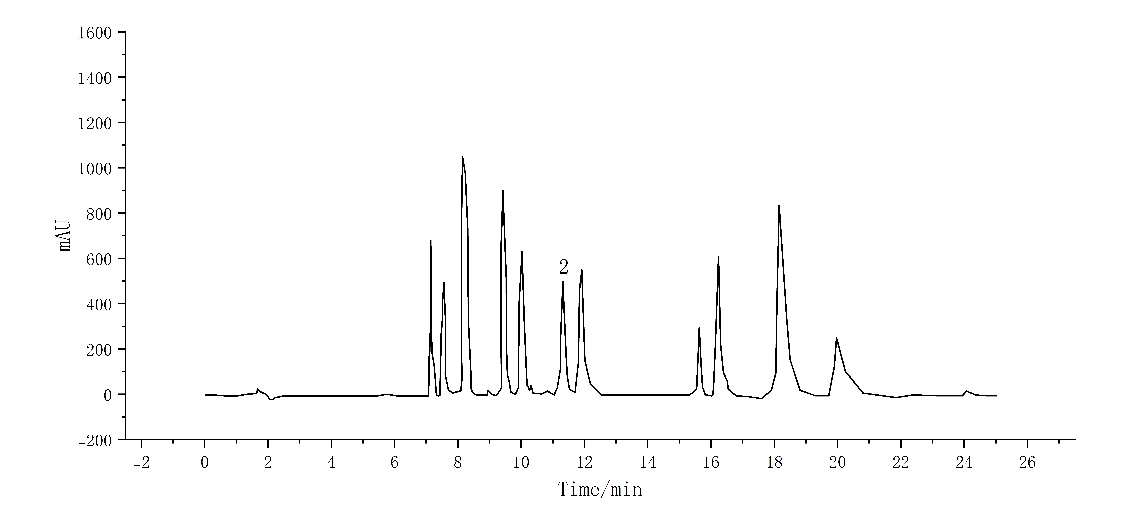


**Figure 6.** Chromatogram of cinnamic acid in roots under Cd20Q2 conditions
